# Patients with Spinal Cord Injuries Favor Administration of Methylprednisolone

**DOI:** 10.1371/journal.pone.0145991

**Published:** 2016-01-20

**Authors:** Christian A. Bowers, Bornali Kundu, Jeffrey Rosenbluth, Gregory W. J. Hawryluk

**Affiliations:** 1 Department of Neurosurgery, University of Utah, Salt Lake City, Utah, United States of America; 2 Department of Physical Medicine & Rehabilitation, University of Utah, Salt Lake City, Utah, United States of America; University of Toronto, CANADA

## Abstract

Methylprednisolone sodium succinate (MPSS) for treatment of acute spinal cord injury (SCI) has been associated with both benefits and adverse events. MPSS administration was the standard of care for acute SCI until recently when its use has become controversial. Patients with SCI have had little input in the debate, thus we sought to learn their opinions regarding administration of MPSS. A summary of the published literature to date on MPSS use for acute SCI was created and adjudicated by 28 SCI experts. This summary was then emailed to 384 chronic SCI patients along with a survey that interrogated the patients’ neurological deficits, communication with physicians and their views on MPSS administration. 77 out of 384 patients completed the survey. 28 respondents indicated being able to speak early after injury and of these 24 reported arriving at the hospital within 8 hours of injury. One recalled a physician speaking to them about MPSS and one patient reported choosing whether or not to receive MPSS. 59.4% felt that the small neurological benefits associated with MPSS were ‘very important’ to them (p<0.0001). Patients had ‘little concern’ for potential side-effects of MPSS (p = 0.001). Only 1.4% felt that MPSS should not be given to SCI patients regardless of degree of injury (p<0.0001). This is the first study to report SCI patients’ preferences regarding MPSS treatment for acute SCI. Patients favor the administration of MPSS for acute SCI, however few had input into whether or not it was administered. Conscious patients should be given greater opportunity to decide their treatment. These results also provide some guidance regarding MPSS administration in patients unable to communicate.

## Introduction

The use of methylprednisolone sodium succinate (MPSS) has been extensively investigated for its putative neuroprotective properties, particularly the reduction in secondary injury that results from acute spinal cord injury (SCI). Following the publication of the second National Acute SCI Study (NASCIS II), MPSS administration was considered a standard of care[1–5]. In recent years, however, MPSS administration for acute SCI has become controversial. Whereas the 2002 Guidelines for the Treatment of Acute SCI recommended MPSS administration at the option level, the 2013 update of these guidelines[6] provides a Level 1 recommendation against the use of MPSS for acute SCI despite little change in the available evidence[6,7,8]. This guideline has been subject to much debate and consensus amongst clinicians has not been achieved on this topic[7, 8].

Proponents of the use of MPSS for SCI argue that the evidence for MPSS administration supports modest neurological benefit although the strength of this evidence is moderate. They argue that when the entire body of literature is viewed as a whole, it more strongly supports MPSS administration than any individual study[1, 5]. Moreover they feel that the small neurological benefits found with MPSS administration are important for SCI patients and justify associated risks. Critics of the administration of MPSS for acute SCI note that MPSS has never demonstrated benefit in the analysis of the primary endpoint in any study[2, 6]. They opine that the statistical methods used in the supporting literature lack rigor and that the potential harms of MPSS outweigh any small potential neurological benefits[2, 6].

In the absence of consensus amongst physicians, it is especially important to consider patient input regarding treatment. Unfortunately, the concomitant injuries that SCI patients may sustain can limit their ability to communicate. Moreover, the time limit for MPSS administration further challenges the ability of physicians to discuss the risks and benefits of MPSS with the patient or substitute decision-maker. As a result of these challenges physicians have historically been paternalistic in their approach to MPSS for SCI. A study documenting the opinions and preferences of SCI patients as it relates to MPSS administration would be helpful, but none has been published to date to our knowledge.

To better understand the opinions and preferences of those with SCI we surveyed patients with SCI whose acute injuries were treated at our institution. All were contacted subsequent to their hospital discharge. We created a brief document plainly summarizing the MPSS literature. We subjected this document to a peer review by 28 SCI experts to ensure it was neutral, free of bias, and of high quality. We then provided it to SCI patients and asked them to complete an online survey after reading it.

## Methods

### Survey Preparation

We reviewed the literature regarding the use of MPSS in the treatment of patients with acute SCI and created a summary sheet outlining the benefits and drawbacks to MPSS use in acute SCI as well as the strength of the evidence (see S1 Appendix). We sent the summary sheet to 28 physicians (neurosurgeons, orthopedic spine surgeons, and critical care physicians), basic scientists, and clinician scientists (see *Acknowledgements*) to adjudicate its quality and to judge whether it exhibited any bias for or against MPSS use in acute SCI. Adjudicators were asked 3 questions and a 5-point Likert scale was used to score their answers. Question1 was, “Is the summary sheet neutral or does it favor or oppose methylprednisolone (MPSS) use for acute SCI?” Question 2 was, “Does the summary sheet demonstrate bias?” and Question 3 asked whether the summary sheet did, “a good job of BRIEFLY summarizing background information on the topic.” *A priori* it was decided that the document would be revised if the median or mode response for Question 1 was not 3, if the median or mode response for Question 2 was >2 or the median or mode response for Question 3 was <4 then the document would be revised and the appraisal would be repeated.

We also created a survey (S2 Appendix) for patients with chronic SCI using the REDCap survey tool which was accessible online. The survey sought background information about the patients’ neurological deficits, the care they received, and their feelings about the care they received. The survey focused on their opinions on the use of MPSS for acute SCI.

### Participants

Participants were identified from two main sources: an electronic database of patients with chronic SCI generated from Physical Medicine and Rehabilitation’s SCI clinic at the University of Utah as well as individuals attending in-person appointments in the outpatient clinic during a two-month time period in early 2015. Patients were eligible if they had received a SCI >3 months prior to the survey period, had been discharged from their acute hospitalization, and if they were older than 18 years of age at the time of survey administration. All respondents provided informed consent for data collection.

All surveys and data acquisition were approved by and in compliance with the regulations of the University of Utah Institutional Review Board (IRB). Data collection and storage was compliant with the Health Insurance Portability and Accountability Act (HIPAA) of 1996. Prior to completion of the survey, all patients had to willfully complete an electronic consent form. They were not permitted to complete the survey unless the consent form was electronically signed. The University of Utah IRB approved of this method of consent.

### Survey Completion and Evaluation

Patients identified through the database were sent an e-mail request for their participation in the completion of an online survey, and patients seen in clinic were given a personal invitation from the medical assistants to complete an online survey on a portable electronic tablet device that was provided. Electronic requests for survey participation were sent out a second time two weeks after the initial request for those who had not responded to the initial electronic study invitation. Patients were asked to complete the survey after reading the MPSS summary sheet that was provided.

### Statistical Analysis

Individual Likert-type item responses were analyzed. Responses were dichotomized into Accept/Agree/Yes and Reject/Disagree/No categories where each category encompassed the top three or bottom three responses. For example, for the question “How important are small neurological improvements from MPSS to you?” the Likert item is scaled 1–10 where 1 is ‘not very important’ and 10 is ‘very important’. Responses 1–3 would be included in one category, and responses 8–10 would be included in the other. Responses were analyzed with the χ^2^ test. Expected outcomes were generated based on the assumption that respondents would have equally selected either category (Agree vs Disagree). Microsoft Excel 2010 and R Statistical Software were used to perform the analyses.

## Results

### Survey Evaluation

**Fig 1:**
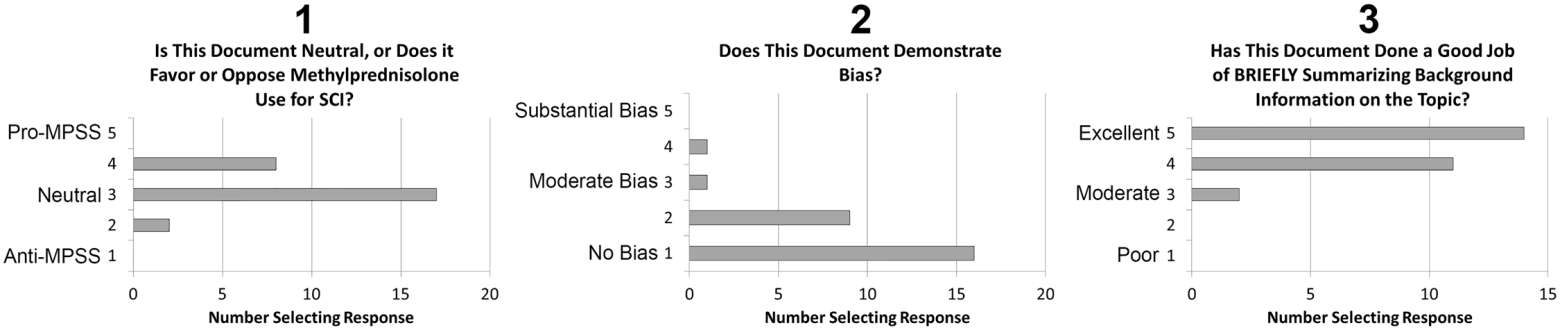
Experts Adjudicated the Datasheet Provided to Spinal Cord Injured Patients as Being Neutral, With Minimal Bias and of High Quality. The authors constructed a datasheet summarizing the literature informing the use of MPSS for SCI. To verify its acceptability prior to distributing to SCI patients we asked twenty eight experts in spinal cord injury to adjudicate the document. The three questions asked of the adjudicators and the frequency of responses is demonstrated above. 27 respondents answered each of the 3 questions. When asked to evaluate whether the information sheet favored (5) or discouraged steroid use (1), the median score was 3 and the mode was 3 (3 = neutral on 5-point Likert scale). Similarly, the median score indicating whether the sheet demonstrated bias was 1 and the mode was 1 (1 = no bias and 5 = extreme bias on a 5-point Likert scale), and the median score for quality of the created survey was 5 and mode was 5 (1 = poor and 5 = excellent on 5-point Likert scale). MPSS = methylprednisolone sodium succinate; SCI = spinal cord injury.

27 of 28 SCI experts who were asked to evaluate the information sheet provided responses. When asked to evaluate whether the information sheet favored (5) or discouraged steroid use (1), the median score was 3 and the mode was 3 (3 = neutral on 5-point Likert scale, Fig 1). Similarly, the median score indicating whether the sheet demonstrated bias was 1 and the mode was 1 (1 = no bias and 5 = extreme bias on a 5-point Likert scale, Fig 1), and the median score for quality of the created survey was 5 and mode was 5 (1 = poor and 5 = excellent on 5-point Likert scale, Fig 1). Based on our pre-specified quality parameters the information sheet was deemed acceptable after this first adjudication and we proceeded to use the document in conjunction with our survey.

### Respondent Characteristics

Of the 384 patients invited to participate, 77 patients completed the questionnaire (20.0%), although some did not answer every question. The vast majority of respondents were male (78%; 57/73). Most reported being paraplegic (58.6%; 41/70) with good arm function: 54.8% (40/73) did not require any assistance with their upper extremities, and 40.5% (30/74) reported full strength with their upper extremities. 16.2% (12/74) patients reported severe upper extremity weakness, and 4.1% (3/74) had complete quadriplegia. Most patients (63%; 46/73) reported arriving to the hospital within 3 hours of their SCI. An additional 19 patients reported arriving within 8 hours of injury. The vast majority of patients (78.7%; 59/75) underwent surgical decompression/stabilization procedures. The majority of patients did not report significant neurological improvement from their initial injury (56% or 42/75 reported a 1–3 on a 10-point Likert scale with 1 as no recovery and 10 as complete recovery).

### Shared Decision-Making Concerning MPSS Treatment

**Fig 2:**
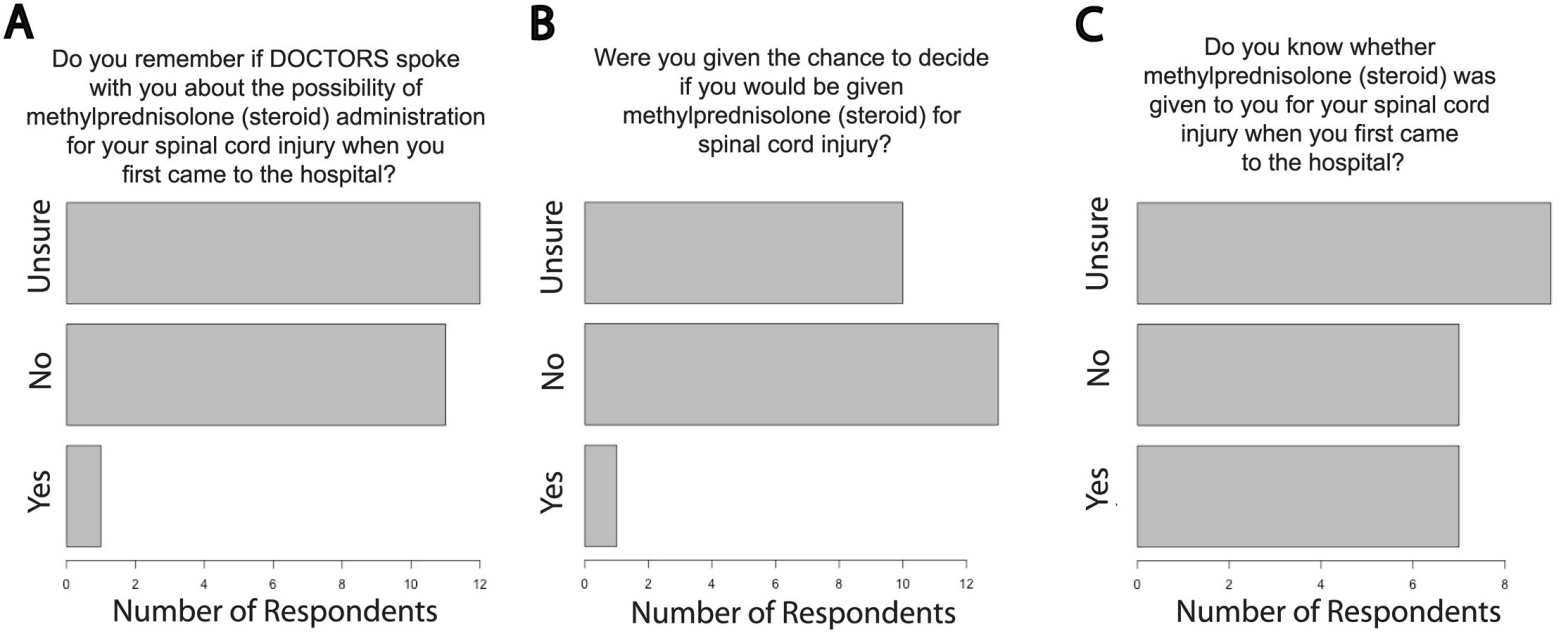
Patients Report Little Input into Decision to Administer Methylprednisolone. Responses of spinal cord injured patients to survey questions related to MPSS discussion and administration. Data reflects the responses of patients who reported presenting to the hospital within 8h of injury conscious and able to talk to the physicians. In panels (**A**), (**B**), and (**C**) the number of respondents choosing each response is plotted. The sample size for (**A**), (**B**), (**C**), were 24, 24, and 23. MPSS = methylprednisolone sodium succinate.

28 (37.3%) respondents indicated being able to converse with their doctors early after injury and of these 17 (60.7%) reported arriving to hospital within 3 hours of injury. An additional 7 (25%) arrived within 8 hours of injury. Of these 24 patients one recalled a physician speaking to them about MPSS and being given the chance to decide whether it should be administered (4.2% for both, Fig 2A). 45.8% of all SCI patients were uncertain if they were treated with MPSS (Fig 2C).

### Respondents’ Opinions Regarding MPSS Use in Acute SCI

**Fig 3:**
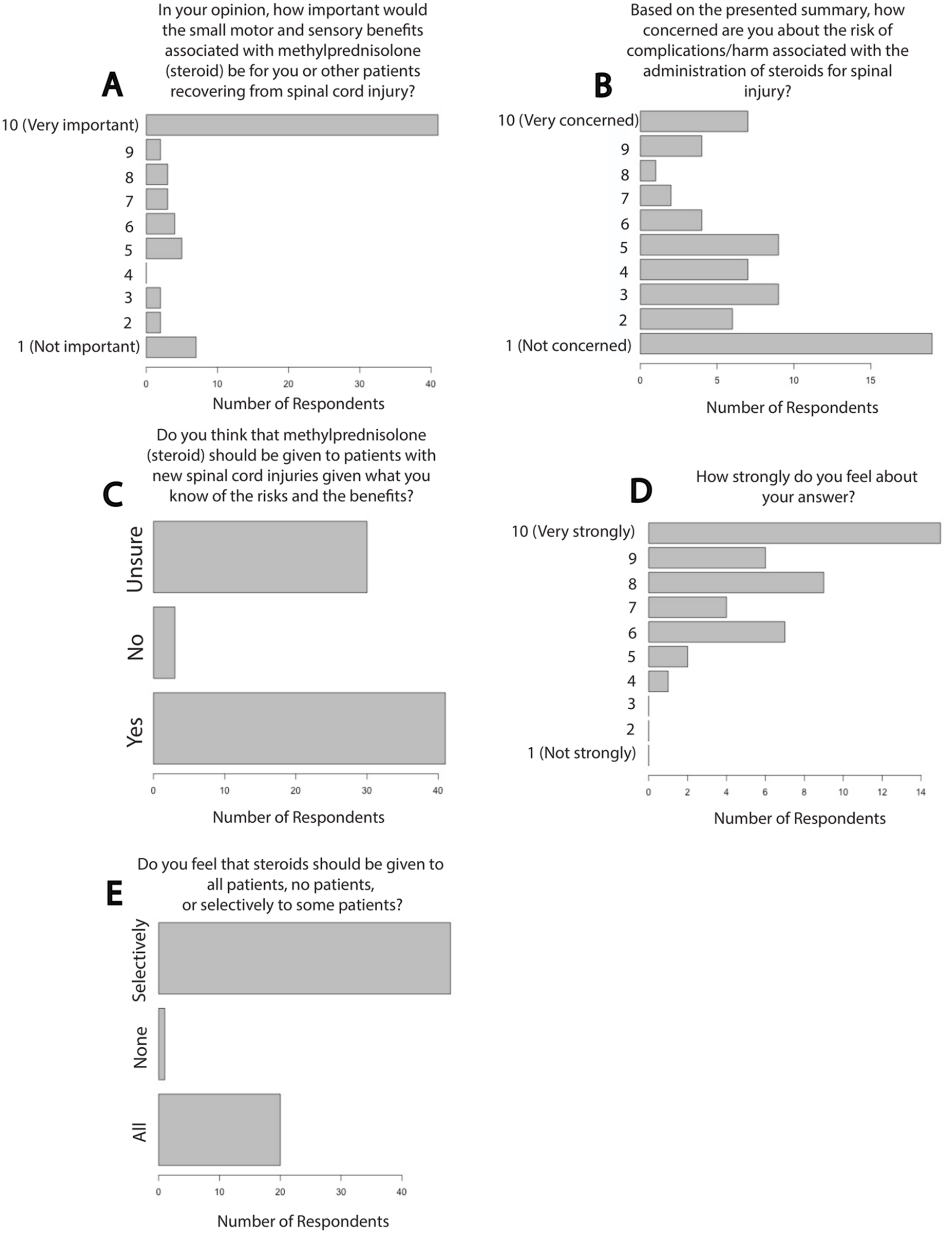
Chronic SCI Patients Favor Administration of Methylprednisolone for Acute SCI. Patient responses to survey questions related to MPSS use for acute SCI are shown. Chi-square testing revealed that responses to all questions differed significantly from expected responses in which all possible answers would have been selected with equal frequency. The sample size for (**A**), (**B**), (**C**), (**D**) and (**E**) were 69, 68, 74, 69 and 44 respectively. MPSS = methylprednisolone sodium succinate, SCI = spinal cord injury.

Most SCI patients expressed that small motor and/or sensory benefits would be important to them: 41 patients (59.4%) selected 10 on a 10-point Likert item with 10 as extremely important (46/69 selected 8–10 on a 10-point Likert scale) and 11 patients (15.9%) reported that small neurological benefits would be unimportant (11/69 selected 1–3 on a 10-point Likert scale; χ^2^ = 21.4, p<0.0001, Fig 3A). 50% of patients had ‘little concern’ for the potential side effects of MPSS (34/68 chose 1–3 on a 10-point Likert scale) while 17.6% of patients were ‘very concerned’ with side effects (12/68, 8–10 on a 10-point Likert scale, p<0.0001, χ^2^ = 10.5, p = 0.001, Fig 3B). 4% of respondents (3/74) believed that MPSS should not be given to new SCI patients given the risks (41/74 reports that MPSS should be given, χ^2^ = 32.8, p<0.0001, Fig 3C). 97.7% of respondents reported feeling strongly about their response as to whether MPSS should be a treatment option for acute SCI patients (30/44 reported a 7–10 on a 10-point Likert scale, χ^2^ = 30.0, p<0.0001, Fig 3D). 29% of patients felt that MPSS should be administered to all patients with acute SCI (20/69), while 1.4% (1/69) felt it should never be administered and 69.6% (48/69) reported a preference for selective administration (testing using all three categories, χ^2^ = 48.6, p<0.0001, Fig 3E).

We explored the possibility that patients who were able to communicate with physicians early after their injury had less severe neurological deficits as a putative explanation for a lack of communication about MPSS. Log-linear analysis suggests that motor function at time of survey, whether the patient could speak at time of presentation, and his or her opinion regarding whether he or she believes patients should receive MPSS are independent (χ^2^ = 23.8, p = 0.36). This provides evidence against such a confound.

## Discussion

Physicians are increasingly using patient surveys to inform management of a variety of pathological conditions ranging from the treatment of Hepatitis C to obesity [9–11]. Patient autonomy is playing a bigger part in medical decision-making, particularly when the “best” treatment is controversial. Surveys of chronic SCI patients have been used to improve the care of those with acute injury. One such study determined that patients preferred to find out about their poor prognosis from a physician soon after their injury[8]. Surveys have also established what SCI patients prioritize in terms of neurologic recovery[9, 10, 12, 13]. To our knowledge, the opinion of SCI patients regarding MPSS administration has not been sought despite the significant controversy surrounding its use following acute SCI.

### MPSS Use For Patients With Acute SCI

The 2002 Guidelines recommend administration of MPSS for acute SCI at the option level; despite minimal changes in the literature informing about the role of MPSS in the treatment of acute SCI the 2013 Guidelines for the Management of Acute SCI produced a Level 1 recommendation to not administer MPSS to patients with acute SCI[6]. This change has been controversial [7, 8]. Amidst this controversy, we contend that physicians have been overly paternalistic in caring for patients with acute SCI, even when the challenges inherent to seeking their opinion on a complex medical decision made under a time constraint is considered. Physicians have largely ignored patient preferences with regards to MPSS administration in the context of acute SCI even though these opinions are of prime importance in all medical decisions. We believe the information garnered by this study will be particularly important for those patients who are unconscious or intubated and unable to express clear opinions.

### Summary of MPSS Literature and Controversy

The NASCIS I, II, and III studies were large, randomized clinical trials (n>500 patients) that examined whether there was any neurological benefit for SCI patients treated with MPSS [14–16]. NASCIS I did not have a placebo group because benefit from MPSS administration was presumed, and thus this trial did not allow the value of MPSS to be assessed as compared with no treatment. Ultimately the doses of MPSS employed in NASCIS I were judged to be too small to confer benefit and it was felt this was a likely explanation for the negative result seen in this study[14]. NASCIS II thus studied the effects of a higher MPSS dose as compared with placebo and demonstrated significantly improved motor function at 1-year follow-up for complete and incomplete patients that received steroids less than 8 hours from injury onset. Here, neurologically incomplete patients benefitted more than complete SCI patients[15]. This result was from a subgroup analysis, thus NASCIS III was specifically designed to compare this result to a higher dosing regimen. Unfortunately, like NASCIS I, NASCIS III did not make comparison to a placebo group as it was judged unethical following NASCIS II. NASCIS III thus does not inform how MPSS administration compares with no treatment. NACISIS III did, however, demonstrate significant improvement for the MPSS group who received MPSS “later” (3–8 hours post-injury) and “longer” (48 hours)[16]. Several additional studies examined the role of MPSS in improving neurological outcomes following SCI but methodological issues prevent strong conclusions from being drawn from these works[17–19]. A more recent large prospective study looking at outcomes with early versus late decompression in cervical SCI identified MPSS administration as being associated with fewer complications[11]. A 2012 Cochrane review showed that NASCIS II MPSS dosing was associated with a significant improvement in motor function at six weeks, six months and when the final measured outcomes were considered, as well as a reduction in mortality (p = 0.15)[5]. When only one-year outcomes were considered the motor benefit barely missed statistical significance (p = 0.066)[5].

An increased risk of complications has been generally seen in studies in which MPSS has been employed for acute SCI. NASCIS I found that the higher dose of MPSS was associated with 3.55 times higher risk of wound infections, 1.65 times higher risk of sepsis and 1.78 times higher risk of DVT/PE though only the risk of wound infections was statistically significant. In NASCIS II steroids were associated with higher rates of wound infection (1.97 times higher gastrointestinal hemorrhage (1.50 times higher) and DVT (3.25 times higher) though none was statistically significant[15]. In NASCIS III the higher MPSS dose was associated with a significant increase in the risk of severe pneumonia (2.23 times higher risk) and a marginally significant increase in the risk of sepsis (1.15 times higher risk)[16]. Nonetheless the 2012 Cochrane review demonstrated that MPSS administration was associated with a trend to improved survival while trends associated with increased risk for gastrointestinal hemorrhage and wound infection did not reach statistical significance[5].

Ultimately, physicians in favor of MPSS for acute SCI feel that SCI is a sufficiently serious condition to merit treatment despite a risk of serious complications similar to the rationale for using chemotherapeutics for malignant disease. Additionally, if adverse events such as infection occur, they can be treated. On the other hand, physicians opposing MPSS use feel the evidence suggesting harm from treatment is stronger than that showing benefit. They feel that statistical conventions were not adhered to in the interpretation of the NASCIS studies. Moreover, they extrapolate the Level 1 evidence against use of steroids in traumatic brain injury to SCI[6].

### Patients’ Opinions Regarding MPSS Use in Acute SCI

We found that a statistically significant number of patients thought that small neurological benefits gained from MPSS would be “very important” and had little concern with possible MPSS-related side effects. These responses were anticipated by our group given that SCI patients are known to seek out therapies which may improve their neurological function such as unproven cellular transplantation therapies[20, 21]. These results suggest the importance that SCI patients place on their neurological function even when the improvements are small. It also highlights the high risk tolerance of these patients. This is helpful information for physicians to consider when treating patients with acute SCI, especially those who cannot communicate on presentation.

### Shared Decision-Making Concerning MPSS Treatment

Shared decision-making is a process during which clinicians and patients collaborate to make health decisions, considering both the best available evidence and patients’ preferences[22]. When controversial treatments divide physicians and when the impact of the condition on the patient is significant, it is even more critical to have shared decision-making with patients regarding their choice of treatment [22]. The controversy regarding MPSS treatment for acute SCI is one such clinical situation. Remarkably, less than 10% of conversant patients presenting to hospital within 8 hours discussed MPSS administration with their physicians or had the opportunity to decide if they would receive it. Furthermore, only 32% knew whether they had received MPSS as part of their acute SCI treatment.

### The Hype Cycle and Selective Administration of MPSS

**Fig 4:**
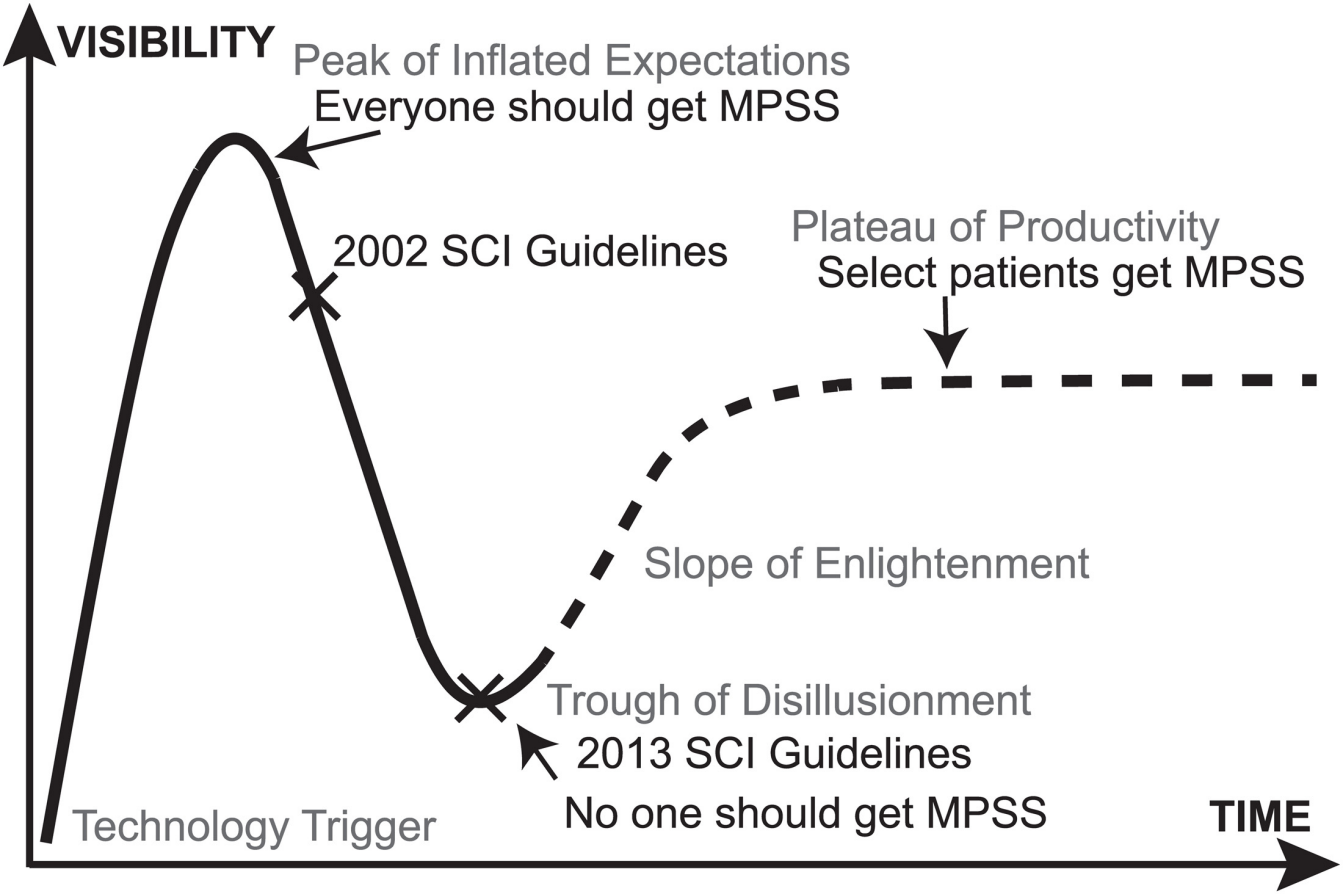
A Hype Cycle of Methylprednisolone for Acute Spinal Cord Injury. The Gartner Hype Cycle describes a common pattern in the adoption of new technologies. Here we propose a Steroid Hype Cycle for Spinal Cord Injury. We contend that the press release of the NASCIS II data in advance of scientific review conforms to a “Peak of Inflated Expectations” while the 2013 Acute Spinal Cord Injury Guidelines are akin to a “Trough of Disillusionment”. This cycle predicts selective administration of methylprednisolone in the future. MPSS = methylprednisolone sodium succinate.

The Hype Cycle (Fig 4) draws from theories of technological innovation and holds that some new ideas and technologies pass through five phases during their dissemination and adoption including (1) technology trigger, (2) peak of inflated expectations, (3) trough of disillusionment, (4) slope of enlightenment and (5) plateau of productivity [23–25]. This Hype Cycle describes a classic pattern of explosive growth when the technology is introduced and viewed as a panacea followed by reduced use as it fails to live up to its initial high expectations. Thereafter usage increases in a more selective, informed fashion until it arrives to a mature “plateau of productivity”. We assert that the use of MPSS for acute SCI conforms to this curve. The “peak of inflated expectations” could be considered the press release that preceded formal publication of NASCIS II, which made MPSS administration—for a time—a standard of care. The 2013 acute SCI guidelines may be considered the “trough of disillusionment”. It is possible that MPSS administration may pass through the “slope of enlightenment” and “plateau of productivity” if selective administration of MPSS is seen in the future. This selective administration is the preference of the authors. We favor MPSS for acute spinal cord injured patients who are young, non-diabetic, exhibit neurological deficits which are of moderate severity and particularly those where the C7 myotome is at risk or potentially salvageable given the importance of the triceps for transferring and independence. Interestingly, spinal cord injured patients reported a preference for selective administration which would presumably see patients with the greatest risk-to-benefit ratio receiving the drug.

### Limitations

This study’s results were obtained from analyses of patients treated at a single institution limiting the generalizability of the findings. Recall bias may also influence the results. We were challenged to provide a data sheet which speaks accurately to the scientific data in the MPSS literature while still making the information understandable to patients. It may be the case that some surveyed patients did not fully understand the summary information, however we think it is unlikely that this substantially altered the responses. Our response rate was lower than we hoped for but a 20% response rate is typical of electronic surveys[7]. We additionally note that recent surveys of spinal cord injured patients fail to report response rates[8, 12, 26, 27] so it is difficult to know how the rate we observed compares with other relevant works. Our survey is vulnerable to selection bias—as most are—because respondent characteristics may differ from those of non-respondents. We anticipate that quadriplegic patients were less likely to respond to our survey and that they may have more strongly favored methylprednisolone administration than paraplegic patients however this is speculative.

## Conclusion

The results of our survey demonstrated that spinal cord injured patients treated at our institution favored having MPSS as a treatment option. After education about the benefits and drawbacks of MPSS, they were largely unconcerned about the possible risks in light of the potential to achieve some neurological improvement. Indeed, they indicated a very strong desire to achieve even small neurological improvements. In light of the disagreements among physicians about the guidelines for the use of MPSS, our results may offer direction to physicians who are managing acute SCI patients, particularly when communication with the patient is not possible. We hope that the lack of shared decision-making demonstrated here will inspire physicians treating patients who have acute SCI to improve communication surrounding the management of this uniquely devastating injury.

## Supporting Information

S1 Appendix Summary of the literature regarding MPSS use for SCI distributed to patients. (DOCX)

S2 Appendix REDCap survey completed by patients. (DOCX)
