# Supplementary material for: Patients with Spinal Cord Injuries Favor Administration of Methylprednisolone
Source: PLoS One. 2016 Jan 20;11(1):e0145991. doi: 10.1371/journal.pone.0145991 (PMC4720442; doi:10.1371/journal.pone.0145991)
Supplement: S1 Appendix — (DOCX) [file pone.0145991.s001.docx]

**Appendix 1: Summary of the literature regard MPSS use for SCI distributed to patients**

**Survey Related to Methylprednisolone Administration for Acute Spinal Cord Injury**

***Principle Investigators:***

*Christian Bowers, MD and Gregory Hawryluk, MD, PhD, FRCSC*

*Department of Neurosurgery, University of Utah*

There is great need for a medicine which can reduce the neurological damage that results from spinal cord injury (SCI). Administration of steroids used to be common practice in SCI but in recent years this has become a very controversial practice[1-5]. The most recently published guidelines state they should not be administered[5]. There is no reported data on the opinion of SCI patients regarding whether MP should be a treatment option. In the hospital it is very hard to get the opinions of patients early after their injuries so for this reason we are asking the opinions of patients with SCIs after their discharge from the hospital. This fact sheet highlights the pros and cons of using steroids for SCI.

Much of the controversy related to steroids for SCI relates to the interpretation of statistics. When a study is completed there is a chance that the results are just a result of luck. A number called a p-value helps doctors and scientists determine when a result is important. The closer a p-value is to zero the less likely the result occurred by chance. Although there is nothing 'magical' about a p-value of 0.05, this is generally considered to be a threshold below which results are important. As well, studies are designed to assess 'primary endpoints' - also known as main results - which consider all patients in a trial. When results come from a subgroup of the examined patients they are considered less valid because the results are more likely to result from chance rather than a real effect.

Three high quality studies (the NASCIS studies[6-8]) were sponsored by the U.S. government to study the potential risks and benefits of steroid treatments for patients with SCI. These trials were conducted over the last three decades. The first study compared two doses of steroids and found no difference in neurological recovery although subsequent studies suggested that both tested doses were too small to be of benefit[6]. This study suggested that the higher steroid doses may increase the risk of complications such as wound infection (3.55 times higher risk with the higher steroid dose) or serious systemic infection (1.65 times higher risk with the higher steroid dose as well as serious blood clots (1.78 times higher risk with the higher steroid dose). Of these risks, only the risk of wound infections met the < 0.05 p-value criteria.

In the second study steroids were associated with a small benefit in motor and sensory functions as compared with placebo. This benefit was only seen in the subgroup of patients who were treated within eight hours of their injury, all having p-values < 0.05[7]. This study suggested that steroids were associated with higher rates of wound infection (1.97 times higher risk with steroids), gastrointestinal hemorrhage (1.50 times higher risk with steroids) and serious blood clots (3.25 times higher risk with steroids), however, none of these risks crossed the 0.05 p-value threshold. This was the only NASCIS study where comparison to a placebo was made and it is therefore the only NASCIS study where we can compare the effect of steroids to no treatment.

In the third study a higher dose of steroids was analyzed and compared to the dose used in the second study[8]. P-values associated with benefit of steroids were slightly larger than 0.05, but were lower than 0.05 in the subgroup of patients treated within eight hours of injury similar to the second NASCIS study. The higher dose was associated with a significant increase in the risk of severe pneumonia (2.23 times higher risk, p< 0.05), a nearly significant increase in the risk of severe systemic infection (1.15 times higher risk, p=0.07). Some other risks were additionally suggested but they did not reach significance. Although some clinicians used the higher dose, many recommended the lower dose as the risk of complications were lower, although so was the neurological benefit.

Several smaller, lower quality studies were additionally performed examining the benefit of steroids for SCI. The highest level of medical evidence comes from a pooled analysis of similar studies. Relevant low and high quality studies were analyzed in such a meta-analysis, performed by the Cochrane Collaboration. The dose used in the second NASCIS study was associated with a nearly significant improvement in motor function (p=0.066)[7]. The overall analysis suggested that steroids were associated with a reduction in mortality (a 46% reduction, p=0.15), an increase in gastrointestinal hemorrhage (a 2.18 times increase, p=0.13) and an increased rate of wound infections (a 2.11 times increase, p=0.13)[4].

Doctors in favor of prescribing steroids for SCI generally feel that although the evidence supporting their use is marginal with respect to traditional conventions, it is sufficiently strong to justify their use. Although the benefit is small (about 10 points of strength on a 112 point scale) they feel that it justifies the suggested increase in the risk of complications. Additionally, they feel that should adverse events such as infection occur, they can be treated. Many also feel that SCI is a sufficiently serious condition to merit treatment with a drug that is of small benefit despite the risk of serious complications.

Doctors who feel that steroids should not be given for SCI feel that a benefit from steroids has never been convincingly shown and that the evidence suggesting harm is stronger than that suggesting benefit. They feel that statistical conventions were not adhered to in the interpretation of the NASCIS studies. They feel that more stringent statistical testing should have been completed and that there were significant flaws in the analysis of the data. They do not feel that small improvements are justified in the context of the risks.

References

1. Bracken, M.B., Methylprednisolone and acute spinal cord injury: an update of the randomized evidence. Spine (Phila Pa 1976), 2001. 26(24 Suppl): p. S47-54.

2. Fehlings, M.G., Summary statement: the use of methylprednisolone in acute spinal cord injury. Spine (Phila Pa 1976), 2001. 26(24 Suppl): p. S55.

3. Chappell, E.T., Pharmacological therapy after acute cervical spinal cord injury. Neurosurgery, 2002. 51(3): p. 855-6; author reply 856.

4. Bracken, M.B., Steroids for acute spinal cord injury. Cochrane Database Syst Rev, 2012. 1: p. CD001046.

5. Hurlbert, R.J., et al., Pharmacological therapy for acute spinal cord injury. Neurosurgery, 2013. 72 Suppl 2: p. 93-105.

6. Bracken, M.B., et al., Efficacy of methylprednisolone in acute spinal cord injury. JAMA, 1984. 251(1): p. 45-52. 7. Bracken, M.B., et al., A randomized, controlled trial of methylprednisolone or naloxone in the treatment of acute spinal-cord injury. Results of the Second National Acute Spinal Cord Injury Study. N Engl J Med, 1990. 322(20): p. 1405-11.

8. Bracken, M.B., et al., Administration of methylprednisolone for 24 or 48 hours or tirilazad mesylate for 48 hours in the treatment of acute spinal cord injury. Results of the Third National Acute Spinal Cord Injury Randomized Controlled Trial. National Acute Spinal Cord Injury Study. JAMA, 1997. 277(20): p. 1597-604.
