# Supplementary material for: Patients with Spinal Cord Injuries Favor Administration of Methylprednisolone
Source: PLoS One. 2016 Jan 20;11(1):e0145991. doi: 10.1371/journal.pone.0145991 (PMC4720442; doi:10.1371/journal.pone.0145991)
Supplement: S2 Appendix — (DOCX) [file pone.0145991.s002.docx]

**Appendix 2: REDCap Survey Completed by Patients**

Survey Questions

1. Are you currently able to walk?
   1. I can walk normally for at least a block without a walker or cane
   2. I can walk normally for only short distances (less than a block) without a walker or cane
   3. I can walk normally for al least a block with a walker or cane
   4. I can walk for only short distances (less than a block) with a walker or cane
   5. I am unable to do anything with my legs
2. At the present time, do you have normal strength in your LEGS?
   1. Full, normal strength
   2. Mild weakness
   3. Moderate weakness
   4. Severe weakness
   5. Unable to move legs at all
3. At the present time, do you require help to do things that you normally do with your ARMS?
   1. No help required
   2. I need a small amount of help to do things with my arms
   3. I need a large amount of help to do things with my arms
4. At the present time, do you have normal strength in your arms?
   1. Full, normal strength
   2. Mild weakness
   3. Moderate weakness
   4. Severe weakness
   5. Unable to move arms at all
5. In your opinion, on a scale from 1-10, home much neurological improvement have you had since your injury?
   1. 1 (none)
   2. 2
   3. 3
   4. 4
   5. 5
   6. 6
   7. 7
   8. 8
   9. 9
   10. 10 (full recovery)
6. Were you conscious and able to talk to the DOCTORS who were treating you when you first arrived at the hospital for treatment of your spinal cord injury?
   1. Yes
   2. No
   3. Unsure
7. To the best of your knowledge, did you arrive at the hospital within THREE hours of the time of your injury?
   1. Yes
   2. No
   3. Unsure
8. To the best of your knowledge, did you arrive at the hospital within EIGHT hours of the time of your injury?
   1. Yes
   2. No
   3. Unsure
9. Do you remember if DOCTORS spoke with you about the possibility of methylprednisolone (steroid) administration for your spinal cord injury when you first came to the hospital?
   1. Yes they did
   2. No they did not
   3. I am unsure if they spoke with me about steroids
10. Were you given the chance to decide if you would be given methylprednisolone (steroid) for spinal cord injury?
    1. Yes
    2. No
    3. Unsure
11. Do you know whether methylprednisolone (steroid) was given to you for your spinal cord injury when you first came to the hospital?
    1. Yes
    2. No
    3. Unsure
12. Were you treated in an intensive care unit (ICU) during the hospitalization for your spinal cord injury?
    1. Yes
    2. No
    3. Unsure
13. Did you require spinal surgery for your spinal injury during your admission for spinal cord injury?
    1. Yes
    2. No
    3. Unsure
14. In your opinion, how important would the small motor and sensory benefits associated with methylprednisolone (steroid) be for you or other patients recovering from spinal cord injury?
    1. 1 (not important)
    2. 2
    3. 3
    4. 4
    5. 5
    6. 6
    7. 7
    8. 8
    9. 9
    10. 10 (very important)
15. Based on the presented summary, how concerned are you about the risk of complications/harm associated with the administration of steroids for spinal injury?
    1. 1 (not concerned)
    2. 2
    3. 3
    4. 4
    5. 5
    6. 6
    7. 7
    8. 8
    9. 9
    10. 10 (very concerned)
16. Do you think that methylprednisolone (steroid) should be given to patients with new spinal cord injuries given what you know of the risks and the benefits?
    1. Yes
    2. No
    3. Unsure
17. How strongly do you feel about your answer?
    1. 1 (not strongly)
    2. 2
    3. 3
    4. 4
    5. 5
    6. 6
    7. 7
    8. 8
    9. 9
    10. 10 (very strongly)
18. Do you feel that steroids should be given to all patients, no patients, or selectively to some patients?
    1. All
    2. None
    3. Selectively
